# Cardiac metastasis in cervical cancer

**DOI:** 10.1259/bjrcr.20150300

**Published:** 2016-05-25

**Authors:** Ajay Sasidharan, Vinod Hande, Umesh Mahantshetty, Shyam Kishore Shrivastava

**Affiliations:** Department of Radiation Oncology, Tata Memorial Hospital, Mumbai, India

## Abstract

Metastasis of cervical carcinoma to the heart is uncommon. Most cases are found during autopsy. These type of metastasis occur mostly in epicardium and myocardium. We present a case report of a patient with carcinoma cervix stage IIIB who presented to the hospital with pitting edema of right lower limb, post 1 year of completion of treatment. PET-CT scan showed FDG avid inguinal, iliac and retroperitoneal lymph nodes, which were bulky on right side causing pedal edema. There was FDG avid uptake seen in right atrial wall and in the atrioventricular groove indicative of metastasis to the heart. Patient refused biopsy or further treatment and hence received best supportive care only. She had a disease free survival of 12 months, and survived for 11 months after being diagnosed with recurrence. Overall survival was 23 months.

## Summary

Cardiac metastasis from cervical cancer is uncommon, with an incidence of 1.23% based on autopsy findings.^1^ Owing to the rarity of the condition, the diagnosis is made almost exclusively post-mortem;^2^ there are few cases of pre-mortem diagnosis and it has been shown that when cardiac metastasis has been found *in vivo,* the prognosis has been extremely poor. A literature search has shown that the longest survival after the diagnosis of cardiac metastasis from cervical cancer has been 13 months.^3^ Aggressive therapy may lengthen patients’ survival and quality of life; but owing to the rarity of the condition, it is very difficult to standardize care for these patients.

The metastatic sites in the heart include the epicardium (60%), myocardium (30%) and endocardium (6%), as well as formation of an intraventricular tumour (3%).^4–8^ Approximately 80% of intracavitary, endocardial or valvular type metastasis occurs in the right chambers of the heart; rarely does metastasis occur in the left chambers. This is attributed to the filtering role of pulmonary circulation and the slower flow in the right chambers.^3^

Here we report a case of carcinoma of the cervix after primary radical treatment with chemoradiotherapy presenting with symptoms suggestive of recurrence that was confirmed on whole-body positron emission tomography (PET)-CT scan that showed recurrence in the pelvic and the para-aortic nodal region, and also in the heart.

**Table 1.** Case reports of cardiac metastasis from cervical cancer cases.

Author	Age (years)	Stage	Type	Primary treatment	Interval to cardiac metastasis (months)	Recurrence diagnosis modality	Pathology confirmation	Recurrence treatment	Cause of death	Time to death from cardiac metastasis (months)	Overall survival (years)
Ando et al^18^	41	IIB	SCC	Sx	8	MRI	Autopsy	CTx	RHF	5	13
Lemus et al^17^	53	IB2	SCC	Sx	14	MRI	Autopsy	CCRT	RHF	1	15
Lemus et al^17^	49	IVB	SCC	ERT	3	MRI and CT scan	None	CCRT	RHF	7	13
Inamura et al^19^	58	IB1	SCC	CTx	44	Echocardiogram and CT scan	Open excision	None	RHF	4	48
Nakao et al^20^	57	IIIB	SCC	CCRT	10	Echocardiogram and CT scan	Open excision	None	RHF	2	12
Borsaru et al^21^	42	IVB	SCC	CCRT	6	Echocardiogram and CT scan	Open excision	-	-	-	-
Kim et al^22^	64	IB1	SCC	CCRT	5	Echocardiogram, TEE and CT scan	Pericardiocentesis	CTx	RHF	7	12
Miller et al^23^	48	IB2	Adeno	CCRT	48	MRI	TEE-guided biopsy	CTx/RT	RHF	8	56
Byun et al^3^	32	IIA	SCC	Sx	15	Echocardiogram and CT scan	Open excision	CTx	Cachexia	13	32
Togo et al^11^	39	IIA	SCC	Sx	23	MRI and CT scan	Biopsy via right IJV	RT	Cardiac tamponde	7	30
Ferraz et al^15^	63	NK	SCC	Sx f/b CCRT	-	CT scan	Open excision	None	-	4	-
Current study	47	IIIB	SCC	CCRT	12	PET-CT scan	-	None	-	11	23

- no available data; Adeno, adenocarcinoma; CCRT, concurrent chemoradiotherapy; ERT, external radiotherapy; f/b, followed by; IJV, internal jugular vein; PET, positron emission tomography; RHF, right heart failure; RT, external radiotherapy; SCC, Squamous cell carcinoma; Sx, surgery; TEE, transoesophagial echocardiogram.

## Clinical presentation

The patient, a 47-year-old obese female, P3L3, postmenopausal for 3 years had initially presented in April 2012 with post-coital bleeding and bilateral lower limb pain. On examination, there was a bulky proliferative growth involving both lips, all fornices and upper one-third of anterior vaginal wall. The left parametrium was medially involved and the right parametrium was involved up to the lateral pelvic wall. Biopsy from the lesion was reported as squamous cell carcinoma of the cervix. Further imaging with MRI of the pelvis, ultrasound scan of the abdomen plus pelvis and X-ray postero-anterior view of the chest was performed. It showed a 4.3 × 5.1 × 5.4-cm mass involving the cervix, with no evidence of hydronephrosis or infiltration into the surrounding organs such as the bladder or the rectum, or grossly enlarged pelvic or para-aortic nodes, and no distant metastasis into the lungs or the liver. No evaluation of any tumour marker was performed. She was diagnosed with International Federation of Gynecology and Obstetrics Stage IIIB cervical cancer (squamous cell carcinoma). She received external beam radiotherapy (EBRT) using 15 MV photons with four-field box technique to a dose of 45 Gy in 25 fractions over 40 days, with a treatment gap of 4 days owing to machine breakdown. She also received five cycles of concurrent chemotherapy of weekly cisplatin at 40 mg m^–2^. After 10 days of EBRT, the patient received high dose-rate intracavitary brachytherapy using the Vienna applicator to a dose of 7 Gy to Point A (2 cm lateral to on either side of tandem, and 2 cm above the ring of the applicator) for four fractions in 1 week. The overall treatment time was 8 weeks.

MRI performed after 3 months showed complete response and the patient was advised for regular follow-up. At the 12-month post-treatment follow-up, she presented with bilateral lower limb pedal oedema of 15 days duration that was associated with pain. There was a history of fever for 3 days, 2 weeks prior to presenting to the hospital. No history of cough, chest or abdominal pain; difficulty in breathing; increased sweating; reduced appetite or weight loss was present. On general examination, a palpable centimetre-sized left supraclavicular lymph node was found. Per vaginal and rectal examination revealed no evidence of disease.

Ultrasonography with colour Doppler of bilateral lower limbs ruled out deep vein thrombosis but showed bilateral lower limb oedema (right > left).

Other than a low haemoglobin level of 9.8 g dl^–1^, other haematological and biochemical investigations did not reveal any significant abnormality. Her electrocardiogram was normal. Owing to normal blood counts and absence of fever, cellulitis was also ruled out.

The PET-CT scan showed increased ^18^F-fludeoxyglucose (^18^F-FDG) uptake in the retroperitoneal, bilateral pelvic and right inguinal nodes and also the presence of disease in the walls of the heart and the aortopulmonary (AP) window lymph node.

Since PET-CT findings showed disseminated disease in a known case of carcinoma of the cervix, the diagnosis of recurrence of carcinoma of the cervix was made. No histological correlation was obtained from the lymph nodes or the cardiac deposits. The deposits in the heart were determined to be metastatic deposits from carcinoma of the cervix based on the PET-CT findings. No two-dimensional (2D) echocardiography or cardiac MRI for further evaluation of these deposits were performed.

## Differential diagnosis for the cardiac deposits

- Metastasis from carcinoma of the cervix;
- atrial myxoma;
- thrombus;
- brown fat;
- mediastinal node;
- variable uptake in a normal heart.

Correlational imaging such as 2D echocardiography of the heart or cardiac MRI is required to differentiate between metastasis, atrial myxoma, thrombus and variable normal uptake in the heart. The diagnosis of metastasis could only be absolutely confirmed with a biopsy or open excision of the lesion. Response to salvage therapy is another way to determine that the lesion is a metastasis from the cervix. Other differential diagnoses such as a mediastinal node and brown fat can be excluded by studying the CT images. For clinical purposes, normal myocardial ^18^F-FDG activity can be defined as absent, or diffusely (with or without some heterogeneity), focally (*e.g.*, papillary muscles) or regionally increased. Knowledge of these normal physiological patterns and the appearance of benign lesions that can mimic malignant disease is important to help differentiate benign from malignant diseases involving the heart.^9^

## Investigations/imaging findings

**Figure 1.**
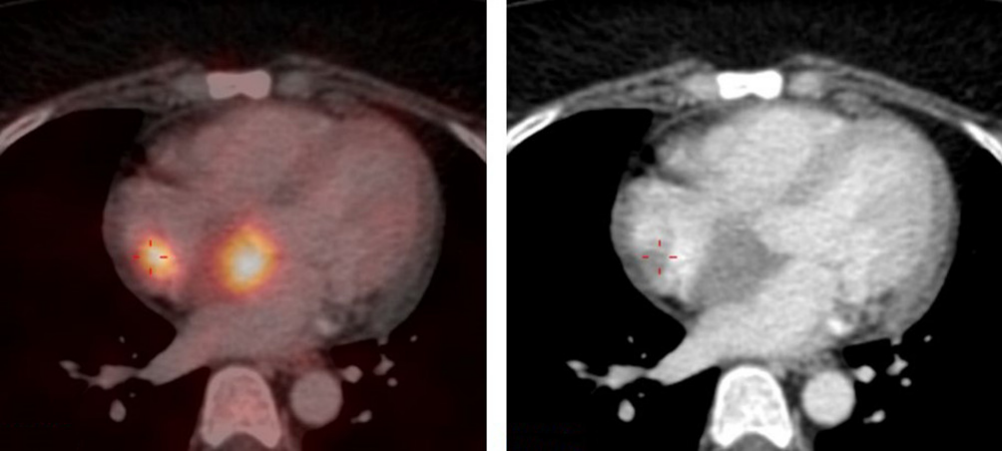
Left: ^18^F-fludeoxyglucose uptake in wall of right atrium and left atrium. Right: CT image showing deposits in the walls of the right and left atrium

**Figure 2.**
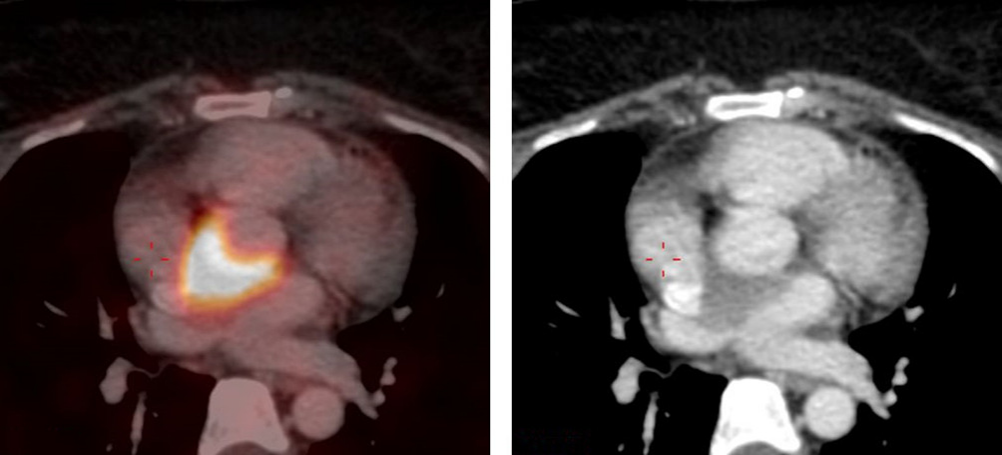
Left: ^18^F-fludeoxyglucose uptake in atrioventricular groove. Right: CT scan showing deposit in the atrioventricular groove.

**Figure 3.**
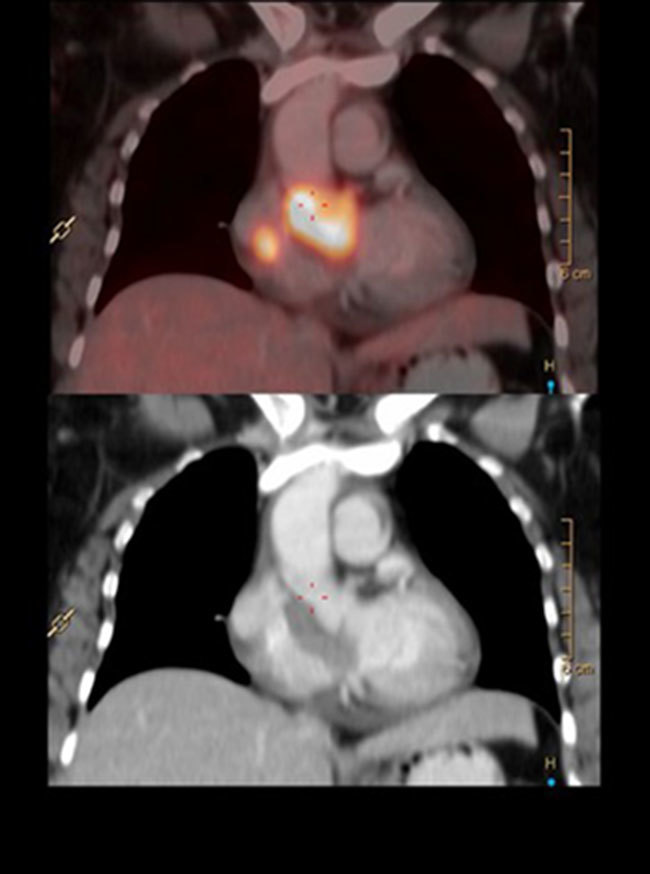
Sagittal sections of positron emission tomography (top) and CT image (bottom) showing cardiac metastasis.

**Figure 4.**
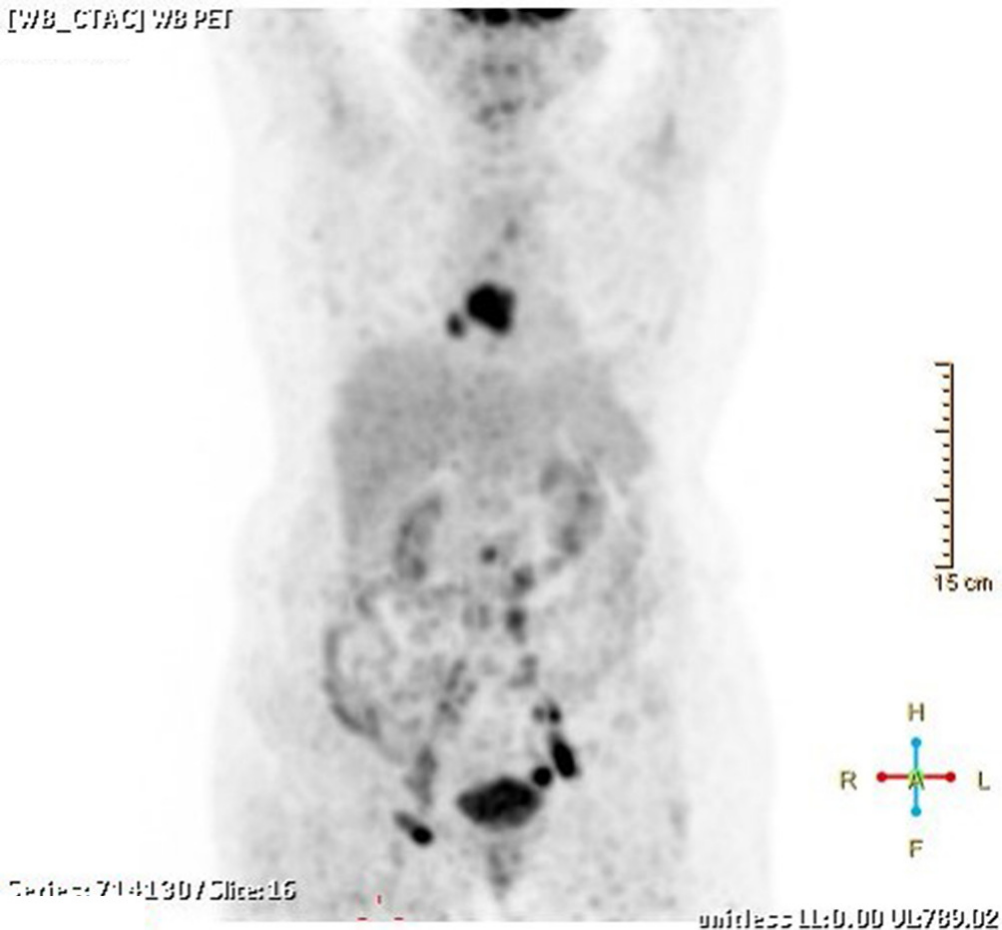
^18^F-fludeoxyglucose uptake in the heart, and para-aortic, bilateral external iliac and inguinal lymph nodes.

PET-CT scan: discrete ^18^F-FDG-avid mass lesions involving the wall of the right and left atrium [maximum standardized uptake value (SUV_max_) 11.8, largest 3.7 × 3.2 cm) (Figure 1). Discrete hypodense ^18^F-FDG-avid lesion in the right atrioventricular groove, measures 30 × 27 mm, SUV_max_ 12.7 (Figure 2). Also seen are discrete ^18^F-FDG-avid enhancing nodes in AP window (largest 12 mm, SUV_max_ 4.8) region. Liver and lungs are unremarkable. Metastatic ^18^F-FDG-avid nodes are noted in following regions: right inguinal (16 mm, SUV_max_ 12.6); bilateral external iliac (largest 27 mm, SUV_max_ 6.4); bilateral common iliac (subcentimetre, SUV_max_ 5.5); multiple retroperitoneal (renal hilar to aortic bifurcation, largest 14 mm, SUV_max_ 7.6). Adnexal structures are otherwise unremarkable (Figures 3 and 4). A 2D echocardiography correlation was suggested for the cardiac deposit.

## Treatment

The patient was given analgesics for her pain and advised to see the pain and the lymphoedema clinic. Medical oncology reference was taken for an opinion on palliative chemotherapy but the patient was not willing to undergo any further investigations or treatment. Hence she was advised best supportive care and follow-up after 6 months.

## Outcome and follow-up

The last follow-up date was March 2014. When contacted over the phone, her relative informed that she had passed away. In this case, the survival after diagnosis of cardiac metastasis was 11 months.

## Learning points

1. Distant metastases of carcinoma of the uterine cervix after treatment commonly affects the lungs, para-aortic nodes, bones, liver, abdominal cavity and supraclavicular lymph nodes. Rarely, it may cause cord compression, especially in the lumbar region, or metastasis to the brain.^10^
2. Cardiac involvement is uncommon; the right ventricle is the most frequently involved, followed by the endocardium. The involvement of the right atrium, as reported in this case, is uncommon and only two cases have been reported in the literature.^11^
3. ^18^F-FDG PET/CT has a sensitivity of 97.5%, specificity of 63.6%, positive-predictive value of 90.9%, and negative-predictive value of 87.5% in detecting recurrence from cervical cancer.^12^
4. Cardiac MRI though is unsurpassed in the evaluation of myocardial anatomy, function and mass.^13^
5. In cases of carcinoma of the uterine cervix, the diagnostic confirmation of cardiac metastasis can also be made using Ga-67 or ^18^F-FDG scintigraphy. The difference between these two methods is that ^18^F-FDG is more sensitive for small lesions.^14^
6. Surgery is rarely necessary, except for establishing a diagnostic line or decompressing pericardial effusions. However, in the presence of an obstructive mass, resection may be helpful in managing invasive and non-invasive tumours.^15^
7. The best results have been obtained in patients whose primary sites had been successfully treated months before or those in whom a total tumour resection could be performed.^16^
8. Radiation therapy to the lesion has also shown good response with symptomatic improvement.^17^ The survival in such cases remains dismal, as all patients develop symptoms again in a matter of months and die of congestive cardiac failure or owing to progression at another metastatic site.

## Consent

Informed consent to publish this case (including images and data) was obtained an is held on record.

## References

[1] Grigsby PW The prognostic value of PET and PET/CT in cervical cancer. Cancer Imaging 2008; 8: 146–55. 18694852 10.1102/1470-7330.2008.0022 PMC2515618

[2] Lam KY, Dickens P, Chan AC Tumors of the heart. A 20-year experience with a review of 12,485 consecutive autopsies. Arch Pathol Lab Med 1993; 117: 1027–31. 8215825

[3] Byun SW, Park ST, Ki EY, Song H, Hong SH, Park JS Intracardiac metastasis from known cervical cancer: a case report and literature review. World J Surg Oncol 2013; 11: 107. 23702302 10.1186/1477-7819-11-107 PMC3667008

[4] Abraham KP, Reddy V, Gattuso P Neoplasms metastatic to the heart: review of 3314 consecutive autopsies. Am J Cardiovasc Pathol 1990; 3: 195–8. 2095826

[5] Butany J, Leong SW, Carmichael K, Komeda M A 30-year analysis of cardiac neoplasms at autopsy. Can J Cardiol 2005; 21: 675–80. 16003450

[6] Klatt EC, Heitz DR Cardiac metastases. Cancer 1990; 65: 1456–9. 2306690 10.1002/1097-0142(19900315)65:6<1456::aid-cncr2820650634>3.0.co;2-5

[7] MacGee W Metastatic and invasive tumours involving the heart in a geriatric population: a necropsy study. Virchows Arch A Pathol Anat Histopathol 1991; 419: 183–9. 1926759 10.1007/BF01626346

[8] Thurber DL, Edwards JE, Achor RW Secondary malignant tumors of the pericardium. Circulation 1962; 26: 228–41. 14037856 10.1161/01.cir.26.2.228

[9] Maurer AH, Burshteyn M, Adler LP, Steiner RM How to differentiate benign versus malignant cardiac and paracardiac ^18^F FDG uptake at oncologic PET/CT. Radiographics 2011; 31: 1287–305. 21918045 10.1148/rg.315115003

[10] Brenner DE Case report carcinoma of the cervix—a review. Am J Med Sci 1982; 284: 31–48. 6283888 10.1097/00000441-198207000-00005

[11] Togo A, Muramatsu T, Tsukada H, Ikeda M, Shida M, Hirasawa T, et al Case of metastatic uterine cervical squamous cell carcinoma in the right atrium. Tokai J Exp Clin Med 2013; 38: 42–5. 23564576

[12] Bhoil A, Mittal BR, Bhattacharya A, Santhosh S, Patel F Role of F-18 fluorodeoxyglucose positron emission tomography/computed tomography in the detection of recurrence in patients with cervical cancer. Indian J Nucl Med 2013; 28: 216–20. 24379531 10.4103/0972-3919.121966 PMC3866666

[13] Scholtz L, Sarkin A, Lockhat Z Current clinical applications of cardiovascular magnetic resonance imaging: review article. Cardiovasc J Afr 2014; 25: 185–90. 25192302 10.5830/CVJA-2014-021 PMC4170175

[14] Shimotsu Y, Ishida Y, Fukuchi K, Hayashida K, Toba M, Hamada S, et al Fluorine-18-fluorodeoxyglucose PET identification of cardiac metastasis arising from uterine cervical carcinoma. J Nucl Med 1998; 39: 2084–7. 9867146

[15] Ferraz JG, Martins AL, de Souza JF, Matos A, Canto AP, Martins AM Metastatic tumor of squamous cell carcinoma from uterine cervix to heart: ante-mortem diagnosis. Arq Bras Cardiol 2006; 87: e104–7. 17128293 10.1590/s0066-782x2006001700025

[16] Poole GV Jr, Meredith JW, Breyer RH, Mills SA Surgical implications in malignant cardiac disease. Ann Thorac Surg 1983; 36: 484–91. 6354118 10.1016/s0003-4975(10)60494-8

[17] Lemus JF, Abdulhay G, Sobolewski C, Risch VR Cardiac metastasis from carcinoma of the cervix: report of two cases. Gynecol Oncol 1998; 69: 264–8. 9648600 10.1006/gyno.1998.5009

[18] Ando K, Kashihara K, Harada M, Kasem I, Nishitani H, Sano N, et al Carcinoma of the uterine cervix with myocardial metastasis. Gynecol Oncol 1997; 65: 169–72. 9103408 10.1006/gyno.1996.4591

[19] Inamura K, Hayashida A, Kaji Y, Ito H, Hirakawa T, Kobayashi H, et al Recurrence of cervical carcinoma manifesting as cardiac metastasis three years after curative resection. Am J Med Sci 2004; 328: 167–9. 15367875 10.1097/00000441-200409000-00006

[20] Nakao Y, Yokoyama M, Yasunaga M, Hara K, Nakahashi H, Iwasaka T Metastatic tumor extending through the inferior vena cava into the right atrium: a case report of carcinoma of the uterine cervix with para-aortic lymph node metastases. Int J Gynecol Cancer 2006; 16: 914–16. 16681785 10.1111/j.1525-1438.2006.00230.x

[21] Borsaru AD, Lau KK, Solin P Cardiac metastasis: a cause of recurrent pulmonary emboli. Br J Radiol 2007; 80: e50–3. 17495056 10.1259/bjr/94870835

[22] Kim HS, Park N-H, Kang S-B Rare metastases of recurrent cervical cancer to the pericardium and abdominal muscle. Arch Gynecol Obstet 2008; 278: 479–82. 18299864 10.1007/s00404-008-0602-y

[23] Miller ES, Hoekstra AV, Hurteau JA, Rodriguez GC Cardiac metastasis from poorly differentiated carcinoma of the cervix: a case report. J Reprod Med 2010; 55: 78–80. 20337214

